# Seasonal Heat Acclimatisation in Healthy Adults: A Systematic Review

**DOI:** 10.1007/s40279-022-01677-0

**Published:** 2022-04-23

**Authors:** Harry A. Brown, Thomas H. Topham, Brad Clark, James W. Smallcombe, Andreas D. Flouris, Leonidas G. Ioannou, Richard D. Telford, Ollie Jay, Julien D. Périard

**Affiliations:** 1 Research Institute for Sport and Exercise (UCRISE), University of Canberra, Bruce, ACT Australia; 2 Thermal Ergonomics Laboratory, Faculty of Medicine and Health, The University of Sydney, Sydney, NSW Australia; 3 FAME Laboratory, Department of Physical Education and Sport Science, University of Thessaly, Trikala, Greece

## Abstract

**Background:**

Physiological heat adaptations can be induced following various protocols that use either artificially controlled (i.e. acclimation) or naturally occurring (i.e. acclimatisation) environments. During the summer months in seasonal climates, adequate exposure to outdoor environmental heat stress should lead to transient seasonal heat acclimatisation.

**Objectives:**

The aim of the systematic review was to assess the available literature and characterise seasonal heat acclimatisation during the summer months and identify key factors that influence the magnitude of adaptation.

**Eligibility Criteria:**

English language, full-text articles that assessed seasonal heat acclimatisation on the same sample of healthy adults a minimum of 3 months apart were included.

**Data Sources:**

Studies were identified using first- and second-order search terms in the databases MEDLINE, SPORTDiscus, CINAHL Plus with Full Text, Scopus and Cochrane, with the last search taking place on 15 July 2021.

**Risk of Bias:**

Studies were independently assessed by two authors for the risk of bias using a modified version of the McMaster critical review form.

**Data Extraction:**

Data for the following outcome variables were extracted: participant age, sex, body mass, height, body fat percentage, maximal oxygen uptake, time spent exercising outdoors (i.e. intensity, duration, environmental conditions), heat response test (i.e. protocol, time between tests), core temperature, skin temperature, heart rate, whole-body sweat loss, whole-body and local sweat rate, sweat sodium concentration, skin blood flow and plasma volume changes.

**Results:**

Twenty-nine studies were included in this systematic review, including 561 participants across eight countries with a mean summer daytime wet-bulb globe temperature (WBGT) of 24.9 °C (range: 19.5–29.8 °C). Two studies reported a reduction in resting core temperature (0.16 °C; *p* < 0.05), 11 reported an increased sweat rate (range: 0.03–0.53 L·h^−1^; *p* < 0.05), two observed a reduced heart rate during a heat response test (range: 3–8 beats·min^−1^; *p* < 0.05), and six noted a reduced sweat sodium concentration (range: − 22 to − 59%; *p* < 0.05) following summer. The adaptations were associated with a mean summer WBGT of 25.2 °C (range: 19.6–28.7 °C).

**Limitations:**

The available studies primarily focussed on healthy male adults and demonstrated large differences in the reporting of factors that influence the development of seasonal heat acclimatisation, namely, exposure time and duration, exercise task and environmental conditions.

**Conclusions:**

Seasonal heat acclimatisation is induced across various climates in healthy adults. The magnitude of adaptation is dependent on a combination of environmental and physical activity characteristics. Providing environmental conditions are conducive to adaptation, the duration and intensity of outdoor physical activity, along with the timing of exposures, can influence seasonal heat acclimatisation. Future research should ensure the documentation of these factors to allow for a better characterisation of seasonal heat acclimatisation.

**PROSPERO Registration:**

CRD42020201883.

**Supplementary Information:**

The online version contains supplementary material available at 10.1007/s40279-022-01677-0.

## Key Points

**Table:** 

Seasonal heat acclimatisation is induced across different climates, from hot and dry to warm and humid.
The adaptations stemming from seasonal heat acclimatisation include reductions in resting core temperature and heart rate, as well as an attenuated rise in core temperature and an increased sweat rate during active and passive heat exposures.
The magnitude of adaptation is dependent on several factors alongside the environmental characteristics, including the timing of environmental exposures during the day, and the duration and intensity of outdoor physical activity.

## Introduction

Environmental heat stress is known to impair aerobic exercise performance [1–3] in response to an increase in whole-body temperature and the consequent adjustments in cardiovascular, central nervous system and skeletal muscle function [4]. The rise in whole-body temperature is also associated with a greater risk of exertional heat illness in uncompensable conditions [5, 6]. However, frequent exposures to hot environments, alongside physical activity, can induce adaptations that attenuate the detrimental effects of environmental heat stress [7–9]. These physiological adaptations occur when thermal stress is sufficient to maintain a disruption of homeostasis to one or more of the biological systems that ensure physiological function and stability during heat exposures [10]. As adaptations develop, decrements in exercise performance are progressively restored [11, 12] and the risk of exertional heat illness reduced [13, 14].

Adaptations to heat stress are referred to as heat acclimation when induced in an artificial setting (e.g. climate chamber) [15–17] and heat acclimatisation when achieved through exposure to a natural environment [18–20]. Much like acclimation, acclimatisation is used to prepare athletes [21, 22] and military personnel [23, 24] for work in hot environments. Both interventions may be purposely implemented to improve thermoregulatory capacity, cardiovascular stability and thermal tolerance during heat exposure [10, 24–26]. However, seasonal heat acclimatisation is largely a background process wherein seasonal changes in ambient conditions can, at least theoretically, induce these same heat adaptations [27]. However, with an increasingly sedentary population [28] and the avoidance of physical activity in warmer parts of the day [29], the magnitude of adaptation induced via seasonal heat acclimatisation in healthy contemporary populations remains unclear.

There is evidence to support the influence of the natural environment inducing heat adaptations during the summer months (i.e. seasonal heat acclimatisation) [30, 31]; however, the reported adaptations differ widely among studies. For example, a greater sweat rate and lower core temperature (*T*_c_) during passive heating were attained following summer (mean maximum ambient temperature 28 °C) in South-Central Japan [30], reinforcing previous results in Japanese athletes using a similar heat response test (HRT) (i.e. 60 and 90 min of lower leg hot-water immersion, respectively) [31]. In contrast, these adaptations were not observed during an incremental running protocol in high-level distance runners following a summer (mean maximum ambient temperature 25 °C) of outdoor running training in the North-Eastern United States [18]. Seasonal heat acclimatisation was also not observed in healthy adults following summer in South-Central Canada, where the environment was described as favourable for inducing heat adaptations (i.e. mean maximum ambient temperature 24 °C) [29]. The differences in seasonal heat acclimatisation suggest that several factors combine to determine the level of heat adaptation attainable during the summer months, in particular the severity of the thermal environment, the accompanying levels of exertion, training status and exposure time. To our knowledge, no systematic review has investigated the level of heat adaptation attainable during summer, while taking into account these factors of influence.

Therefore, the aim of this review was to systematically evaluate the available literature regarding the impact of seasonal heat acclimatisation in healthy adults on markers of heat adaptation, including a lower absolute *T*_c_, enhanced sweat rate, and lower heart rate for a given absolute exercise intensity. By characterising the magnitude and factors that influence seasonal heat acclimatisation, athletes, coaches and policy makers will be able to make more informed decisions regarding performance and safety in the heat.

## Methods

The data analysis and inclusion criteria were documented before the systematic review and registered with the PROSPERO International prospective register for systematic reviews website (CRD42020201883).

### Data Sources

A comprehensive electronic search using the databases MEDLINE, SPORTDiscus, CINAHL Plus with Full Text, Scopus and Cochrane was conducted on 9 July 2020. The final electronic search took place on 15 July 2021. The search strategy included first and second-order search terms. The first-order keywords were ‘acclim*’ and ‘adapt*’. These were used in conjunction with the second-order search terms, ‘heat*’ and ‘season*’. Search terms were piloted to ensure a comprehensive identification of relevant articles. When available, searches were limited to full-text journal articles written in the English language that focused on human subjects. The search strategy used to identify relevant seasonal heat acclimatisation articles can be found in Supplementary material Tables S1, S2 and S3 in the Electronic Supplementary Material (ESM).

### Study Selection

Abstracts, reviews and unpublished theses were excluded. Following implementation of the search strategy, potential references were imported into Covidence (Covidence systematic review software, Veritas Health Innovation, Melbourne, Australia), reference lists were screened for additional papers and duplicates were removed, and one review author verified these duplicates. One author scanned the titles and abstracts for relevance and progressed studies to the full-text stage if they met the following inclusion criteria: English language, human participants, assessed outcome variables associated with heat adaptation, and tested the same healthy adult participants (≥ 18 years of age) pre-and post-season (i.e. summer). Studies with < 3 months between HRT were deemed insufficient to measure seasonal adaptations and were excluded from the review. Additionally, studies were excluded if participants relocated to conduct a hot weather training camp as this is not representative of seasonal acclimatisation. The full-text screening was undertaken by two authors, disagreements were discussed, and a third author made the final decision if no agreement was reached.

### Outcome Measures

Following a full-text review, data were extracted from relevant studies for the following variables: participant age, sex, body fat percentage, body mass, height, maximal oxygen uptake ($\dot{V}$O_2max_), time spent exercising outdoors (i.e. intensity, duration, frequency), environmental conditions during summer (i.e. mean ambient temperature, mean relative humidity), HRT (i.e. protocol, time between tests), *T*_c_ (i.e. oesophageal, rectal, tympanic, sublingual), skin temperature (*T*_sk_), heart rate, whole-body sweat loss, whole-body and local sweat rate, sweat sodium concentration, skin blood flow and plasma volume changes. Data for *T*_c_, *T*_sk_ and heart rate were extracted at rest to determine the effect of seasonal heat acclimatisation on the change (Δ) in these variables, as well as during the HRT to determine the difference in the change in these variables following acute heat exposure (e.g. smaller increase in *T*_c_, *T*_sk_ and heart rate). Data were extracted from two time points from each study (i.e. pre–post summer, start–end of summer or summer–winter).

In addition to the extraction of environmental conditions provided by the studies, this review sought to provide a mean daytime wet-bulb globe temperature (WBGT) for the summer months specific to each included study. Only one study provided WBGT [32]. For the remaining studies, environmental data including air temperature, relative humidity, wind speed and cloud coverage were obtained from the National Oceanic and Atmospheric Administration (http://www.ncei.noaa.gov/data/global-hourly). The obtained wind speed values were adjusted for height above the ground and air friction coefficient using published methodology [33]. Solar radiation was computed for the location of each study [34], while accounting for cloud coverage [35]. Thereafter, mean daytime (08:00 to 18:00) WBGT values were calculated throughout the summer using the approach described by Liljegren et al. [36], which is the recommended method to use when calculating WBGT from meteorological data [37]. These methods have previously been used to assess the heat acclimatisation state of individuals undergoing physical work in a laboratory study [38], and to estimate the heat stress experienced by athletes in ecological studies [39, 40].

### Risk of Bias Assessment and Data Extraction

Two reviewers independently assessed the risk of bias for the included studies using the McMaster critical review form for quantitative studies [41], which was modified by Chalmers et al. [42] (ESM Table S4). The critical review form classifies each domain as possessing either high, low or unclear risks of bias. A customised data extraction template was piloted to ensure its suitability. Two review authors extracted data using this template, disagreements were discussed, and if no agreement was reached, a third author made the final decision. The authors extracted mean values, standard deviations (SD) and sample sizes. If a standard error was reported, it was converted to SD, and where studies did not directly report mean values, these were visually estimated from figures and verified by an additional author. Review Manager (Version 5.4, The Cochrane Collaboration, 2020) was used to calculate mean differences and 95% confidence intervals (CI), using mean, SD and sample size.

### Data Synthesis

Findings from the 29 included studies that matched the inclusion criteria are presented in accordance with PRISMA (Preferred Reporting Items for Systematic Reviews and Meta-Analyses) guidelines [43]. Environmental conditions, frequency and duration of heat exposure, and physical activity have all been shown to influence heat adaptation, and due to heterogeneity within these factors, a meta-analysis was not conducted. Instead, this review describes the studies and the details that could impact the magnitude of seasonal heat acclimatisation in relation to the outcome variables identified.

## Results

### Study Selection

**Fig. 1:**
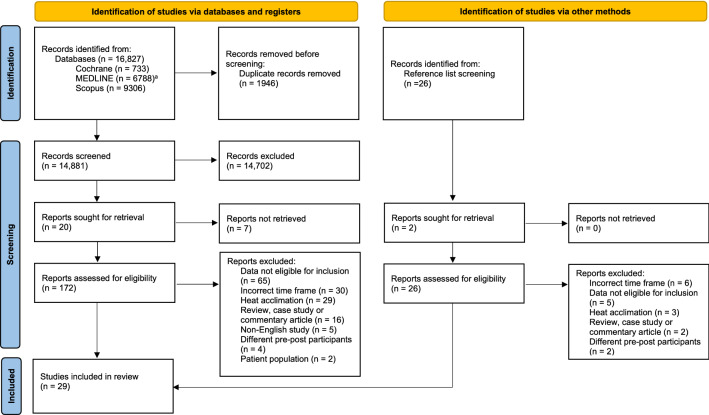
PRISMA flow diagram for study inclusion process. ^a^MEDLINE (EBSCOhost)—includes SPORTDiscus and CINAHL Plus

Figure 1 highlights the process for study inclusion. Initial electronic searches and reference screening returned 16,853 potential studies. Of the electronic search results, 1946 were duplicates and 14,702 were deemed irrelevant and thus removed. This left 205 studies for full-text eligibility screening, after which a further 176 studies were excluded (Fig. 1). Reasons for exclusion included too short of intervention [44–46], participants being relocated to a warmer climate [47], mean data could not be determined [48–51] and the retesting of the same population sample was unclear [52–59]. The final data synthesis included 29 studies.

### Risk of Bias

For all studies included in the review, a clearly outlined study purpose and an appropriate conclusion were provided. The most frequent risk of bias related to the sample of participants. Two studies lacked comprehensive documentation of descriptive statistics for their sample [60, 61], and all but two studies [29, 32] failed to justify their sample size. One of these studies included a retrospective power analysis showing an insufficient sample size was not the reason for the lack of heat adaptation in their population, which helped strengthen their conclusions [29].

Reducing the inferential power of seasonal heat acclimatisation studies was the lack of a control group, as reported within a review on short-term heat acclimation [42]. Given that the implementation of a control group where environmental exposure is minimised is impractical, a potential solution may be to configure groups based on the level/dose of heat exposure encountered (e.g. athletes versus office workers). The risk-of-bias score for each study is included in Table 1.

**Table 1:** Participants, heat response test protocols, Köppen-Geiger climate classification, reported summer exposure duration and risk of bias of the 29 included studies

References	Participant characteristics (*n*, sex)	Heat response test and conditions (°C, %RH, m·s^−1^)	Testing time frame	Köppen–Geiger classification	Summer exposure duration	McMaster risk of bias (**–**/8)
Araki et al. [79]	11F—trained 8F—untrained	120 min cycling at 160 W (30, 60, 0.3)	Summer–winter	Humid subtropical climate		6
Armstrong et al. [18]	4M, 1F	90 min running at 80–200 m·min^−1^ (30, 35, 4.5)	Pre-post summer	Warm-summer humid continental	Endurance training 25 days·month^−1^; 83% noon and onwards	5
Bain and Jay [29]	8M	90 min cycling at 60% $\dot{V}$O_2max_ (22, 37, < 0.1)	Pre-post summer	Warm-summer humid continental	4.6 h·week^−1^ outdoor PA	6
Bates and Miller [75]	29M	35 min cycling at 40% $\dot{V}$O_2max_ (35, 50, ‘minimal’)	Summer–winter	Hot-summer Mediterranean climate		5
Benjamin et al. [32]	25M	60 min running at 59% $\dot{V}$O_2peak_ (35, 48, 1.8)	Pre-post summer	Warm-summer humid continental	6 h·week^−1^ outdoor training	6
Buguet et al. [66]	4M, 4F	24-h record following normal routine (–, –, –)	Pre-post summer	Hot semi-arid climate	3–4 h·day^−1^ weekdays; 6–10 h over weekends	4
Doupe et al. [73]	72M	30 min rest (20, 40–50, **–**)	Summer–winter	Warm-summer humid continental		4
Finberg and Berlyne [81]	4M	30 min cycling at $\dot{V}$O_2_ of 1.2 L·min^−1^ (50, 11, **–**)	Summer–winter	Hot semi-arid climate		4
Finberg et al. [60]	5M	90 min walking at 4.7 km·h^−1^ (50, 15–23, < 0.1)	Summer–winter	Hot semi-arid climate		6
Gold et al. [76]	17M	Successive levels of PA (40, 42, **–**)	Summer–winter	Hot semi-arid climate		4
Hori [31]	15M—athlete 15M—control	30 min passive heating + 90 min leg HWI 42 °C (30, 70, **–**)	Winter–summer	Humid subtropical climate		3
Hori and Tanaka [83]	12M	30 min passive heating + 30 min cycling at 50% $\dot{V}$O_2max_ (30, 60, **–**)	Summer–winter	Humid subtropical climate		4
Ihzuka et al. [72]	42M—Okinawa 44M—Mainland	60 min leg HWI 42 °C (30, 70, **–**)	Summer–winter	Humid subtropical climate		4
Inoue et al. [30]	6M—young 8M—old	60 min leg HWI 42 °C (30, 45, **–**)	Summer–winter	Humid subtropical climate		5
Keatisuwan et al. [82]	14F	20 min passive heating + 60 min cycling at 40% $\dot{V}$O_2max_ + 30 min passive heating (40, 30, < 0.2)	Winter–summer	Humid subtropical climate		5
Lee et al. [67]	15M	30 min leg HWI 42 °C (25, 60, < 1.0)	Summer–winter	Humid continental hot summers with dry winters	≥ 10 h·week^−1^	5
Lei et al. [70]	12M	60 min cycling at 40% $\dot{V}$O_2max_ (32, 75, 0.2–1.1)	Winter–summer	Humid subtropical climate		5
Li and Tokura [80]	6F—skirts 6F—trousers	60 min passive heating (37, 30, 0.5)	Pre-post summer	Humid subtropical climate		6
Lui et al. [63]	12M—WLFF 12M—control	60 min walking at 50% $\dot{V}$O_2max_ (43, 33, **–**)	Pre-post summer	Cold semi-arid climate	600 h over summer 200 h over summer	6
Matsumoto et al. [71]	6M	30 min leg HWI 43 °C (25, 60, **–**)	Winter–summer	Humid subtropical climate		4
Notley et al. [65]	12M, 2F—young 9M, 1F—old	180 min passive heating (44, 30, **-**)	Winter–summer	Warm-summer humid continental	9 h·week^−1^ PA 7 h·week^−1^ PA	6
Shapiro et al. [78]	8M	50 min walking at 1.34 m·s^−1^ + 10 min passive heating + 50 min walking at 1.34 m·s^−1^ (40, 30, 1.0)	Summer–winter	Humid continental hot summers with year around precipitation		5
Shin et al. [68]	15M	QSART (24, 40, < 1.0)	Summer–winter	Humid continental hot summers with dry winters	≥ 10 h·week^−1^	5
Shvartz et al. [19]	11M	90 min walking at 5.6 km·h^−1^ (50, 20, 0.4)	Winter–summer	Hot semi-arid climate		3
Taniguchi et al. [69]	5M 7F	160 min passive progressive heating (up to 42, 40, **–**)	Summer–winter	Humid subtropical climate		6
Torii and Nakayama [77]	4M	20 min cycling at 40% $\dot{V}$O_2max_ (30, 45, 1.0)	Winter–summer	Humid subtropical climate		2
Torii et al. [61]	3M	20 min bed scale cycling at 40% $\dot{V}$O_2max_ (30, 45, **–**)	Winter–summer	Humid subtropical climate		5
Umemiya [74]	7M	Up to 120 min rest (26, 50, **–**)	Summer–winter	Humid subtropical climate		3
Zhang et al. [64]	15M, 15F—NV 15M, 15F—SAC	60 min passive heating (32, 50, **–**)	Summer–winter	Humid subtropical climate	M: 3.5 h·week^−1^ PA F: 2.5 h·week^−1^ PA	6

Skirts: item of clothing worn over summer. Trousers: item of clothing worn over summer. McMaster Risk of Bias was scored out of 8 with higher scores indicating a lower risk of bias

*F* female, *HWI* hot water immersion, *M* male, *NV* naturally ventilated, *PA* physical activity, *QSART* quantitative sudomotor axon reflex test, *RH* relative humidity, *SAC* split air conditioners, $\dot{V}$*O*_*2max*_ maximum oxygen consumption, *WLFF* wildland firefighter

### Individual Study Results

There were 561 participants included in the selected studies, 90 (16%) of which were female. Weighted mean age (26 ± 10 years, *n* = 400), height (169.7 ± 8.8 cm, *n* = 392), body mass (63.4 ± 12.1 kg, *n* = 430) and $\dot{V}$O_2max_ (49 ± 11 mL·kg^−1^·min^−1^, *n* = 128) were extracted from 21, 20, 22 and 9 studies, respectively. The 29 studies provided information for seasonal heat acclimatisation from eight countries (Japan *n* = 13, Israel *n* = 4, United States *n* = 4, Canada *n* = 3, South Korea *n* = 2, Australia *n* = 1, China *n* = 1, Niger *n* = 1). Within these eight countries, the Köppen–Geiger climate classification system identified that 22 of the 29 studies were conducted in humid environments: humid subtropical climate (Cfa) *n* = 14, Hot-summer humid continental climate (Dfa) *n* = 1, Warm-summer humid continental climate (Dfb) *n* = 5, and Humid continental hot summers with dry winters (Dwa) *n* = 2 [62]. Mean daytime summer WBGT for the included studies was 24.9 °C (SD 3.1 °C, median 26.0 °C), ranging from 19.5 °C [63] to 29.8 °C [64] (Table 2).

**Table 2:** Mean daytime environmental data for the summer months between 08:00 and 18:00 across the 29 included studies

References	Dry-bulb temperature (°C)	Black globe temperature (°C)	Relative humidity (%)	Wind speed (m·s^−1^)	Wet-bulb globe temperature (°C)
Araki et al. [79]	25.2	31.9	73.4	0.9	24.9
Armstrong et al. [18]	22.5	30.9	56.3	1.7	21.1
Bain and Jay [29]	20.6	26.3	68.3	2.5	19.7
Bates and Miller [75]	26.5	30.5	40.9	2.9	21.3
Benjamin et al. [32]	24.3	35.4	64.4	1.3	24.4
Buguet et al. [66]	36.9	44.7	23.4	3.1	27.8
Doupe et al. [73]	20.5	27.0	66.4	2.3	19.6
Finberg and Berlyne [81]	28.4	41.8	43.9	1.1	26.3
Finberg et al. [60]					
Gold et al. [76]	29.0	41.3	44.1	1.4	26.4
Hori [31]	29.7	38.8	64.0	1.1	28.6
Hori and Tanaka [83]	27.5	36.8	61.5	1.0	26.4
Ihzuka et al. [72]	28.9	37.8	72.8	1.3	28.7
Inoue et al. [30]	26.6	37.8	71.6	1.0	27.2
Keatisuwan et al. [82]	26.7	32.7	68.2	0.9	25.5
Lee et al. [67]	26.4	32.4	70.2	1.3	25.4
Lei et al. [70]	27.1	32.7	71.7	1.2	26.0
Li and Tokura [80]	21.3	30.7	55.0	0.5	20.6
Lui et al. [63]^a^	24.0	30.3	36.8	2.0	19.5
Matsumoto et al. [71]	26.8	34.2	72.8	1.3	26.4
Notley et al. [65]	23.4	29.8	60.0	1.8	21.6
Shapiro et al. [78]	23.6	29.7	62.1	1.3	22.0
Shin et al. [68]	27.3	35.1	64.9	0.9	26.2
Shvartz et al. [19]					
Taniguchi et al. [69]	27.9	36.3	61.1	0.5	26.7
Torii and Nakayama [77]	29.3	37.3	66.0	0.8	28.2
Torii et al. [61]	26.7	32.6	68.5	2.3	25.3
Umemiya [74]	29.1	35.7	62.9	1.1	27.2
Zhang et al. [64]	31.2	38.0	68.2	1.0	29.8

^a^Environmental data that heat acclimatisation group were exposed to in Montana, USA

Table 1 provides an overview of the selected studies, including environmental information about the study location, exposure duration and a risk-of-bias score. Nine (31%) studies provided information regarding the environmental exposure of their participants via a training diary/self-reported physical activity [18, 29, 32, 64, 65], a work schedule [63] or an estimation of exposure time from day-to-day life [66–68]. Reported physical activity was greatest in the studies investigating endurance athletes, with ~ 6 h·week^−1^ of outdoor physical activity [32] and 25 days·month^−1^ of endurance training [18]. Two studies reported physical activity from 2.5 to 9 h·week^−1^ but did not differentiate between indoor and outdoor physical activity [64, 65], whilst four studies estimated outdoor exposure without reporting physical activity [63, 66–68] (Table 1). One study asked participants to remain sedentary between the summer and winter HRT [69], whilst another [70] instructed participants to limit outdoor physical activity and training to ≤ 3 sessions per week before summer testing.

The most common method for assessing seasonal heat acclimatisation was via an active HRT (*n* = 16). Ambient conditions for these tests ranged from temperate (22 °C, 37% relative humidity [RH] [29]) to hot-dry (50 °C, 22% RH [19]) and more humid environments (32 °C, 75% RH [70]). Alongside the range of environmental conditions was a broad spectrum of exercise protocols, ranging from 20 min of recumbent cycling at 40% $\dot{V}$O_2max_ [61], to 90 min of upright cycling at 60% $\dot{V}$O_2max_ [29]. An additional nine studies implemented passive HRT, with the majority (*n* = 5) utilising lower leg hot-water immersion for either 30 [67, 71], 60 [30, 72] or 90 min [31]. Experimental protocols for the remaining studies included resting in temperate conditions [73, 74], quantitative sudomotor axon reflex testing (QSART) [68], or 24-h data recording during regular daily routines [66]. The implementation of each protocol was tailored to both the purpose and the participants within each study. For example, Armstrong et al. [18] conducted an incremental active HRT in well-trained distance runners to assess their level of seasonal heat acclimatisation via changes in *T*_c_, sweat rate and *T*_sk_. In contrast, Zhang et al. [64] assessed thermal comfort in college-aged students by identifying the most comfortable resting conditions inside an environmental chamber. These examples highlight the differences in methodologies of the included studies, thus reducing the capacity to draw firm conclusions.

**Fig. 2:**
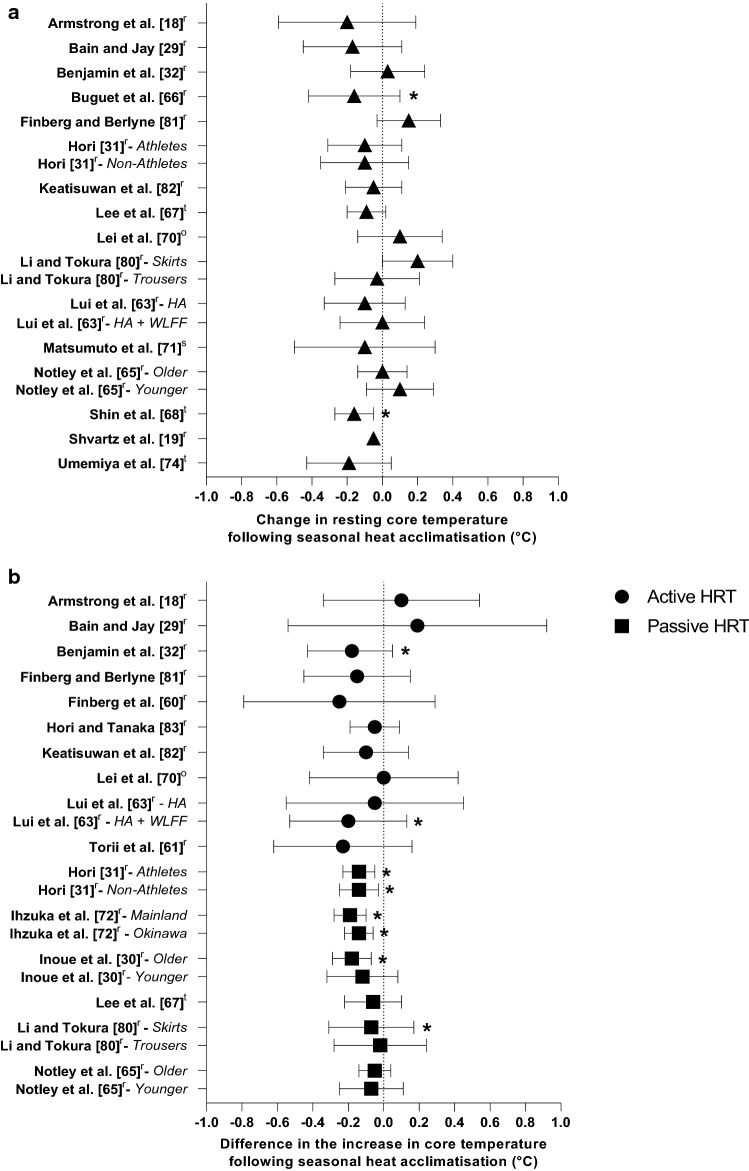
Change in core temperature at rest (**a**) and difference in the increase in core temperature during active and passive heat response tests (HRT) (**b**) following seasonal heat acclimatisation. Data are presented as mean with 95% confidence intervals (CIs). Study without 95% CIs did not report variability of the mean. *HA* heat acclimatisation, ^o^ Oesophageal temperature, ^r^ Rectal temperature, ^s^ Sublingual temperature, ^t^ Tympanic temperature, *WLFF* wildland firefighter. *Significant effect of seasonal heat acclimatisation (*p* < 0.05)

The extent of data available for extraction ranged from a maximum of five outcome variables in one study, to some instances where only one outcome variable was available. Twenty studies provided data for at least one outcome variable that was measured at rest (*T*_c_, *T*_sk_, heart rate and plasma volume). The majority (*n* = 21) of studies included *T*_c_ data (Fig. 2a and b). The remaining eight studies either had no *T*_c_ measure [64, 73, 75, 76], were influenced by pre-cooling [77], or the change in *T*_c_ could not be determined [69, 78, 79]. Of the 16 studies that reported a resting *T*_c_, mean results ranged from 36.36 to 37.51 °C pre-summer and 36.20 to 37.40 °C post-summer. However, resting measures were not standardised for the time of day between studies and may have been influenced by circadian rhythm [66]. The change in *T*_c_ during acute heat exposure could be calculated for 11 studies that implemented an active HRT and a further six studies using a passive HRT. Of these 17 studies, six [30–32, 63, 72, 80] reported a 0.1–0.2 °C reduction in the change in *T*_c_ during heat exposure following seasonal heat acclimatisation (Fig. 2b).

**Fig. 3:**
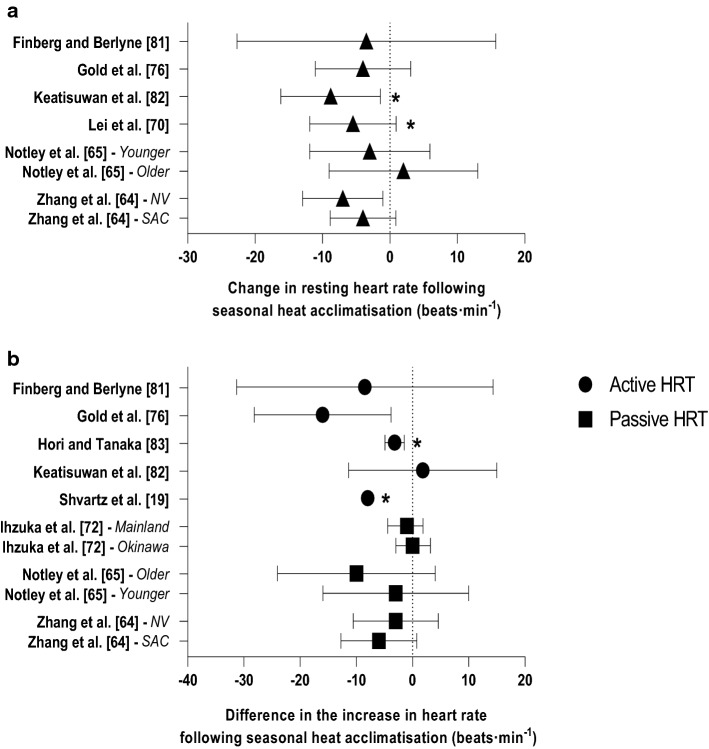
Change in heart rate at rest (**a**) and difference in the increase in heart rate during active and passive heat response tests (HRT) (**b**) following seasonal heat acclimatisation. Data are presented as mean with 95% confidence intervals (95% CIs). Study without 95% CIs did not report variability of the mean. *NV* naturally ventilated, *SAC* split air conditioners. *Significant effect of seasonal heat acclimatisation (*p* < 0.05)

Six studies reported resting heart rate [64, 65, 70, 76, 81, 82]. Mean resting heart rate ranged from 75 to 80 beats·min^−1^, while following seasonal heat acclimatisation, mean resting heart rate was 69 to 75 beats·min^−1^. Two groups recorded the reduction in resting heart rate to be significant (Fig. 3a) [70, 82]. The largest reduction (9 beats·min^−1^) was recorded in a group of healthy females who also recorded the highest pre-acclimatisation resting heart rate (80 ± 9 beats·min^−1^) [82]. Two of the five active HRT assessing the change in heart rate during exercise documented reductions of up to 8 beats·min^−1^ [19, 83]. This is a similar trend to that seen during passive HRT, but none reached statistical significance (Fig. 3b; *n* = 3) [64, 65, 72].

**Fig. 4:**
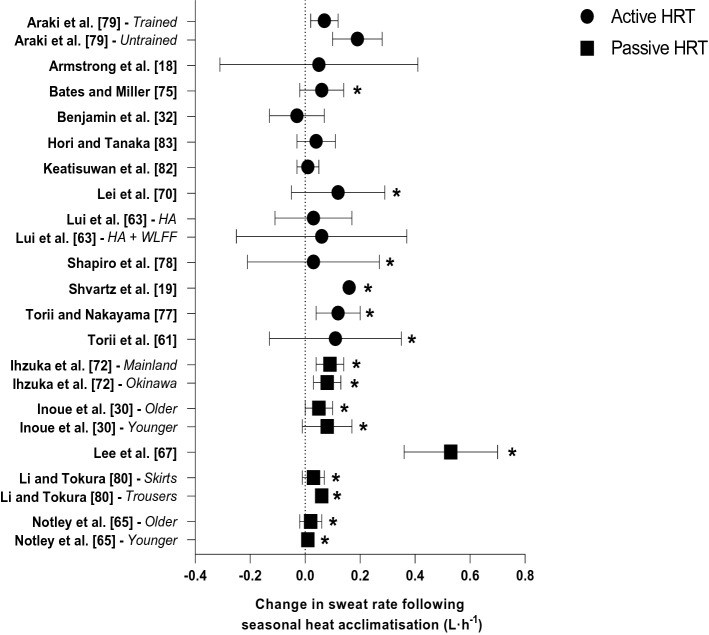
Change in sweat rate during active and passive heat response tests (HRT) following seasonal heat acclimatisation. Data are presented as mean with 95% confidence intervals (95% CIs). Study without 95% CIs did not report variability of the mean. *HA* heat acclimatisation, *WLFF* wildland firefighter. *Significant effect of seasonal heat acclimatisation (*p* < 0.05)

Thirteen studies measured whole-body or local sweat rate during exercise but only six reported an increase following seasonal heat acclimatisation [19, 61, 70, 75, 77, 78]. The increase in whole-body sweat rate during active HRT ranged from 0.03 [78] to 0.16 L·h^−1^ [19]. Conversely, all studies that utilised a passive HRT and recorded whole-body sweat rate (Fig. 4) observed an increase (*n* = 5), with the largest increase (0.53 L·h^−1^) recorded in a group of healthy males [67]. Seasonal heat acclimatisation led to increases in the percentage of body mass lost due to sweat secretion during both active [70] and passive [31, 67, 72, 80] HRT. The largest increase was a percentage change in body mass loss of 89%; however, this only equated to an absolute change in body mass loss of 67 g [80]. The remaining studies reported increases in body mass loss of 140 g [31], 70 g [72] and 267 g [67].

For several outcome variables, only a small number of studies contributed to the systematic review (skin blood flow *n* = 4 and resting plasma volume *n* = 1). Two studies [30, 65] reported changes in forearm skin blood flow via venous occlusion plethysmography in mL·100 mL^−1^ tissue·min^−1^, with an additional two studies using laser Doppler flowmetry and reporting their data as a percentage of maximum [29] or arbitrary units [70]. Irrespective of the testing method, no statistically significant changes were reported in forearm skin blood flow following seasonal heat acclimatisation. Lei et al. [70] and Notley et al. [65] recorded skin blood flow at second sites (back and calf, respectively). While no significant changes were recorded at the back, seasonal heat acclimatisation led to a significant increase in calf skin blood flow in younger adults and a significant decrease in older adults at the start of a passive HRT. No significant changes were recorded during the passive HRT [65]. The one study to assess resting plasma volume reported an 8% expansion following summer in Winnipeg, Canada [73].

**Fig. 5:**
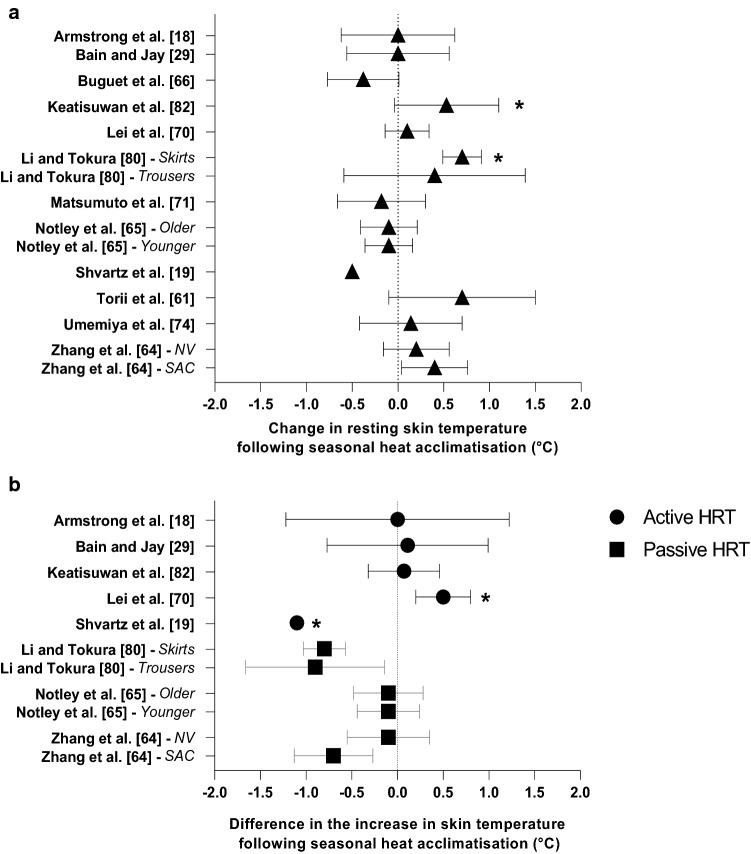
Change in skin temperature at rest (**a**) and difference in the increase in skin temperature during active and passive heat response tests (HRT) (**b**) following seasonal heat acclimatisation. Data are presented as mean with 95% confidence intervals (95% CIs). Study without 95% CIs did not report variability of the mean. *NV* naturally ventilated, *SAC* split air conditioners. *Significant effect of seasonal heat acclimatisation (*p* < 0.05)

Based on the available data, it appears that the change in climate across summer induces seasonal heat adaptations. The environmental data extracted from the studies indicate that a range of climates (Table 2) can induce seasonal heat acclimatisation (Figs. 2, 3, 4, 5). For example, a significant reduction in resting *T*_c_ following summer was observed in two studies with an estimated WBGT of 27.0 °C. The reductions in resting heart rate and resting *T*_sk_ were reported following a mean summer WBGT of 25.8 °C (*n* = 2) and 23.1 °C (*n* = 2), whilst plasma volume expansion was documented following a mean summer WBGT of 19.6 °C (*n* = 1). The reductions in the change (i.e. smaller increase) in *T*_c_, *T*_sk_ and heart rate during the HRT were evident following a mean summer WBGT of 25.9 °C (*n* = 5), 26.0 °C (*n* = 1) and 26.4 °C (*n* = 1), respectively. Increases in sweat rate and reductions in sweat sodium concentration were reported following a mean summer WBGT of 25.1 °C (*n* = 11) and 26.3 °C (*n* = 6), respectively. Collectively, these data suggest that seasonal heat acclimatisation in one or several physiological responses corresponds with a mean summer WBGT of 25.2 °C (SD 2.8 °C, median 26.0 °C, *n* = 19).

## Discussion

The aim of this systematic literature review was to characterise the magnitude of physiological heat adaptation occurring in response to seasonal heat acclimatisation. The findings indicate that physiological adaptations can be induced during summer across various climates, but the magnitude is highly variable and likely influenced by environmental conditions, exposure duration and time of day, as well as the intensity and duration of outdoor physical activity. The combination of these factors also influences the magnitude of adaptation between variables.

### Core Temperature

Of the 16 studies that reported resting *T*_c_, 11 documented a reduction after summer (range: − 0.05 to − 0.20 °C) [18, 19, 29, 31, 63, 66–68, 71, 74, 82], although statistical significance was reached in only two studies [66, 68]. Both studies reported a 0.16 °C reduction in resting *T*_c_ following comparable environmental exposure durations (≥ 10 h·week^−1^) in environments with a mean summer WBGT of 26.2 °C [68] and 27.8 °C [66]. However, the manner in which time was spent during the environmental exposures was unclear and physical activity (i.e. intensity, duration) was not reported. Therefore, it is difficult to determine how these factors, or their interaction, mediated the reductions in resting *T*_c_.

Nine studies provided data regarding environmental exposure duration [18, 29, 32, 63–68], with the highest being in wildland firefighters [63]. These firefighters were not only exposed to the Western United States summer (i.e. Colorado, New Mexico, Montana), but also to additional radiative heat emitted from fire. Compared with the non-firefighters who were only exposed to the summer months, they were exposed to heat for ~ 400 more hours [63]. While the firefighters experienced no resting *T*_c_ reduction, they exhibited the largest reduction in the change in *T*_c_ (0.2 °C) during an active HRT post-summer, possibly due to an earlier onset threshold for sweating [63]. Although this provides insight into the seasonal heat acclimatisation of firefighters, the nature of their heat stress (i.e. exposure to high levels of radiant heat) is different to that of many occupations and presents an added difficulty when quantifying their heat exposures.

Finberg et al. [60] observed a larger reduction in the change in *T*_c_ (0.25 °C) during a 90-min treadmill walk, but it did not reach statistical significance, likely due to low statistical power (*n* = 5). This was also the case for the reported increase in sweat rate (0.10 kg·m^−2^·h^−1^) and reductions in end-exercise heart rate (10 beats·min^−1^) [60]. The authors did not document physical activity or environmental exposure duration during the study, but did report a mean ambient temperature of 30.3 °C in the midday shade during summer [60]. This location (i.e. Beer-Sheva, Israel) has previously been shown to induce seasonal heat acclimatisation in untrained males (i.e. greater sweat rate, reduced *T*_sk_ and heart rate during exercise) [19].

Of the six studies that utilised passive HRT, four documented a smaller increase in *T*_c_ following summer (Fig. 2b) [30, 31, 72, 80]. Three of the four utilised hot-water immersion and assessed *T*_c_ using a rectal thermistor [30, 31, 72]. A fifth study reported a similar magnitude in the change in *T*_c_ (− 0.1 °C) during hot-water immersion using tympanic temperature, which was not statistically significant [67]. The non-significant result may be due to the variable nature of tympanic temperature measurement, which is acknowledged as being less accurate than other *T*_c_ measurement techniques (e.g. oesophageal) [84].

### Skin Temperature

The change in *T*_sk_ during HRT was assessed in eight investigations, with three (all active HRT) noting an effect of seasonal heat acclimatisation [19, 32, 70] (Fig. 5b). Following a summer in Beer-Sheva (Israel), which has a semi-arid climate, untrained males completed a 90-min walk in dry conditions (50 °C, 20% RH) and recorded a 1.1 °C reduction in the change in *T*_sk_ [19]. In this instance, however, the lower *T*_sk_ was not associated with a lower *T*_c_ [19]. In contrast, others have reported a 0.5 °C higher *T*_sk_ during a humid (75% RH) HRT in summer, but this did not lead to a lower *T*_c_ [70]. However, as highlighted by the authors, sweating efficiency is reduced in humid climates due to a lower vapour pressure gradient between the skin and the environment, thus requiring an elevation in *T*_sk_ to promote dry heat loss [70].

### Sweat Rate and Sensitivity

Studies utilising a passive HRT all documented an increased sweating rate after summer [30, 65, 67, 69, 71, 72, 80], except for a group of mainland Japanese residents [72] and one group of untrained males [69]. However, when the authors of the latter study plotted local sweat rate against mean body temperature, the onset and thermosensitivity of sweating became statistically significant for the untrained males [69]. In the studies utilising an active HRT, an earlier onset threshold for sweating was observed following seasonal heat acclimatisation [18, 19, 61, 63, 79], as well as enhanced sweating thermosensitivity [70, 78], and up to a 63% increase in whole-body sweat rate [19, 61, 70, 75, 77, 78]. This increase in sweat rate, however, was only noted in four participants during 20 min of cycling at 40% $\dot{V}$O_2max_ post-summer, corresponding to an increase of 0.12 L·h^−1^ in relation to a whole-body sweat rate of 0.31 L·h^−1^ [77]. Other studies have reported increases of 0.13 and 0.16 L·h^−1^, noting whole-body sweat rates following summer of 0.83 L·h^−1^ [70] and 1.35 L·h^−1^ [19], respectively. The greatest absolute increase in sweat rate was reported in healthy young males (0.53 L·h^−1^) following a minimum outdoor exposure duration of 10 h·week^−1^ over the summer months [67]. The authors also reported an earlier onset threshold for sweating during the post-summer hot-water immersion test [67]. However, during an identical HRT in a similar population, an earlier onset threshold for sweating was not observed, while total body sweat rate increased [30]. Although the environmental conditions were similar between these two studies (i.e. summer WBGT 25.4 °C [67] and 27.2 °C [30]), the occasional participation in sporting activities described by Inoue et al. [30] is unlikely to have matched the level of activity undertaken by the participants in the Lee et al. [67] study.

Interestingly, Inoue et al. [30] also investigated seasonal heat acclimatisation in a group of older males (aged 60–65 years), noting that their enhanced sudomotor capacity (i.e. an increased total body sweating rate) was induced more slowly and decayed more quickly compared with a younger group (aged 20–25 years). A more recent investigation has also reported greater whole-body and local sweat rates following seasonal heat acclimatisation in older adults (aged 55–72 years), but the results were similar to a group of younger adults (aged 19–27 years) [65]. Both studies assessed physically active older adults, and whilst Inoue et al. [30] described their participants as active, no specific level of activity was provided [30]. In contrast, Notley et al. [65] indicated that their participants completed ~ 7 h·week^−1^ of moderate-to-vigorous physical activity, which helps in the interpretation and characterisation of seasonal heat acclimatisation across populations.

Whole-body sweat rate is dependent on a number of factors, including heat acclimatisation status [7], metabolic heat production [85], age [30], sex [86], environmental conditions [85, 87] as well as aerobic capacity [88]. Araki et al. [79] investigated different exercise intensities and their influence on whole-body sweat rate. The authors reported that trained females have an earlier onset threshold for sweating compared with untrained females when exercising at the same absolute work rate, regardless of the season. Interestingly however, whole-body sweat rate did not increase in the trained females following summer [79], a result similar to that of wildland firefighters [63], well-trained runners [18], and endurance athletes [32]. Perhaps the regular exercise undertaken by these trained individuals provides partial heat adaptation [89, 90], which seasonal heat acclimatisation failed to further enhance. However, the high $\dot{V}$O_2max_ and regular training of the well-trained runners and endurance athletes may not have been the primary factor precluding the increase in whole-body sweat rate [18, 32], but rather the environmental conditions each group were exposed to (i.e. summer WBGT 21.1 °C [18] and 24.4 °C [32]), which may not have been severe enough to exacerbate thermal strain and induce thermal adaptation. In addition, the upper physiological limits of heat dissipation (i.e. maximal skin wettedness) may not have been challenged under compensable heat stress conditions, hindering the likelihood of observing an improvement in heat dissipation (i.e. increased whole-body sweat rate) [91]. Thus, it appears that additional research is required in sufficiently stressful environments (i.e. ambient temperature, relative humidity, solar radiation, wind speed) to elucidate the effect of seasonal heat acclimatisation on sudomotor adaptations in well-trained individuals.

### Sweat Sodium Concentration

Sweat sodium concentration decreased (19–59%) in six studies during active and passive HRT following seasonal heat acclimatisation [30, 31, 72, 75, 82, 83]. The reduction of sodium in sweat helps maintain plasma osmolality, which has been shown to prevent sweat rate reductions encountered when an individual becomes hypohydrated during prolonged exercise in the heat [92]. It is also suggested that a more dilute sweat facilitates evaporation by widening the water vapour gradient between the skin and the environment, as electrolytes (i.e. sodium, chloride) lower the water vapour pressure at the level of the skin for a given temperature [10]. The studies documenting a reduction in sweat sodium concentration examined healthy individuals [30, 31, 72, 82, 83] and outdoor workers [75]. In contrast, both Armstrong et al. [18] and Benjamin et al. [32] examined endurance athletes and did not observe a reduction in sweat sodium concentration following summer. The lack of adaptation may relate to the high $\dot{V}$O_2max_ (~ 69 mL·kg^−1^·min^−1^) and regular training of the runners, particularly in the Armstrong et al. [18] study, which may have provided partial heat adaptation, as evidenced by an already low baseline sweat sodium concentration (21 mmol·L^−1^). It is also noteworthy that both the testing site (i.e. limb, trunk or whole-body) and analytical technique can impact the measurement of sweat sodium concentration [93]. Armstrong et al. [18] and Benjamin et al. [32] used the whole-body washdown technique, which has been suggested to be the most precise [93], whereas the other studies used regional measures. Notwithstanding, seasonal heat acclimatisation appears to decrease sweat sodium concentration, with the magnitude of adaptations potentially influenced by aerobic fitness and training habits.

### Cardiovascular Stability

Heart rate at rest [70, 82] and during exercise in the heat [19, 32, 83] has been shown to decrease following seasonal heat acclimatisation. During exercise at the same absolute work rate, heart rate was 3–8 beats·min^−1^ lower in summer compared with winter [19, 83]. The reduction in heart rate during exercise is considered a hallmark heat adaptation, which along with plasma volume expansion leads to an increased stroke volume and better maintained cardiac output [94]. Several integrative mechanisms support the improvement in cardiovascular stability (e.g. reduced heart rate) stemming from regular heat exposure [7], including the expansion of plasma volume [95], which has been shown to undergo seasonal fluctuations [73]. However, as the latter study [73] did not investigate seasonal heat acclimatisation, heart rate was not recorded and the impact of seasonal plasma volume expansion on the heart rate response was not assessed. Other factors, such as a reduction in resting *T*_c_, also contribute to attenuate the elevation in *T*_c_ and thus heart rate during exercise in the heat [4], further highlighting the integrative nature of heat adaptations on the improvements in cardiovascular function.

Within the studies utilising an active HRT [18, 19, 29, 32, 60, 61, 63, 70, 75–79, 81–83], firm conclusions regarding the influence of seasonal heat acclimatisation on cardiovascular function are difficult to draw, as environmental exposure durations were not provided. Although smaller increases in heart rate during passive HRT were noted following summer, none were statistically significant [64, 65, 72]. However, when data were pooled from seven different conditions (i.e. 20–32 °C and 50–70% RH) during a comparison of individuals residing in naturally ventilated or air conditioned environments, a significantly smaller increase in heart rate during the post-summer passive HRT was observed [64]. For the most part, these data indicate that heart rate at rest and during exercise in the heat is reduced following summer.

## Considerations for Future Research

Seasonal heat acclimatisation has been assessed in different climates, from warm-humid [29] to hot semi-arid [66] (Table 1). While the reporting of environmental conditions may not have been within the scope of several studies, it is recommended that future studies provide a detailed account of the climate when evaluating seasonal heat acclimatisation. For example, environmental conditions for Ottawa, Canada were provided in depth, including mean daily minimum and maximum air temperature for the entire testing period of one study [29]. Moreover, nine studies (31%) provided information regarding participant physical activity. Benjamin et al. [32] combined in-depth descriptive statistics for exercise training and meteorological data, using nearby meteorological observation stations and training devices (i.e. Garmin, Polar), allowing for the calculation of WBGT and heat index for each individual outdoor training session. An improved quantification of environmental exposure may allow for a comparison of groups based on exposure levels, potentially providing minimum environmental exposure thresholds for the inducement of seasonal heat acclimatisation.

This review focused on healthy adults with 434 of the 524 participants being males. Three papers focused on heat adaptation in females across different seasons [79, 80, 82], whereas 19 studies exclusively investigated males. Given the thermoregulatory differences between sexes (i.e. females demonstrating a lower maximum sweat rate) [96] and different rates of heat adaptation between males and females [97, 98], a greater representation of female participants is required. The need to investigate additional populations further extends to well-trained athletes and outdoor workers. Moreover, to our knowledge, no seasonal heat acclimatisation research has been conducted in child and adolescent populations. It has been suggested that children may adapt to the heat more slowly and to a smaller magnitude than adults, thus placing them at a thermoregulatory disadvantage during exercise in hot environmental conditions [46, 99]. Numerous heat acclimatisation guidelines have been created to enhance the safety of children and adolescents during exercise in the heat [13, 14, 100]. However, seasonal heat acclimatisation is yet to be assessed in these populations and is required given the potential differences in the development of adaptations.

## Conclusion

Despite variation in outcomes related to variables indicative of seasonal heat acclimatisation, the weight of evidence suggests that seasonal heat acclimatisation is induced across different climates (e.g. hot and dry, warm and humid). The physiological adaptations reported include reductions in resting *T*_c_ (0.16 °C) and heart rate (range: 5–9 beats·min^−1^), an attenuated increase in *T*_c_ (range: 0.10–0.20 °C), reduced sweat sodium concentration (range: − 22 to − 59%) and an increased sweat rate (range: 0.03–0.53 L·h^−1^) during active and passive heat exposures. These adaptations were associated with a mean summer WBGT of 25.2 °C (range: 19.6–28.7 °C). However, the magnitude of adaptation developing during the summer months is dependent on several factors alongside the environmental characteristics, including the timing of environmental exposures during the day, as well as the duration and intensity of outdoor physical activity. These factors all play pivotal roles in the development of seasonal heat acclimatisation and should be recorded and reported in future research.

## Supplementary Information

Below is the link to the electronic supplementary material.

Supplementary file1 (DOCX 30 kb)

## Declarations

### Availability of data and material

Additional data can be provided on reasonable request to authors.
