# Supplementary material for: Seasonal Heat Acclimatisation in Healthy Adults: A Systematic Review
Source: Sports Med. 2022 Apr 23;52(9):2111–28. doi: 10.1007/s40279-022-01677-0 (PMC9388416; doi:10.1007/s40279-022-01677-0)
Supplement: Supplementary file 1 — Supplementary file1 (DOCX 30 kb) [file 40279_2022_1677_MOESM1_ESM.docx]

**Article title:** Seasonal heat acclimatisation in healthy adults: A systematic review

**Journal name:** Sports Medicine

**Authors:** Harry A. Brown^1^, Brad Clark^1^, Thomas H. Topham^1^, James W. Smallcombe^2^, Andreas D. Flouris^3^, Leonidas G. Ioannou^3^, Richard Telford^1^, Ollie Jay^2^, Julien D. Périard^1^

Author affiliations: ^1^Research Institute for Sport and Exercise (UCRISE), University of Canberra, Bruce, ACT, Australia

^2^The University of Sydney, Thermal Ergonomics Laboratory, Faculty of Medicine and Health, Sydney, NSW, Australia

^3^FAME laboratory, Department of Physical Education and Sport Science, University of Thessaly, Trikala, Greece

**Corresponding author:** Julien D. Périard

University of Canberra, Research Institute for Sport and Exercise Science, Bruce, ACT, Australia

Email: Julien.Periard@canberra.edu.au

Phone: +61 (0)2 6206 8540

**Supplementary material**

**Table S1.** Search Strategy.

| **Database:** MEDLINE (EbscoHost) with CINAHL Plus with Full text, SPORTDiscus with Full Text | |
| --- | --- |
| **Order** |  |
| 01 | Acclim* |
| 02 | Adapt* |
| 03 | Heat* |
| 04 | Season* |
| 05 | 01 or 02 |
| 06 | 03 or 04 |
| 07 | 05 and 06 |
| 08 | 05 and 06. Limiters – Full Text |
| 09 | 05 and 06. Limiters – Full text. Narrow by language - English |

Table S2. Search Strategy.

| **Database:** Scopus | |
| --- | --- |
| **Order** |  |
| 01 | Acclim* |
| 02 | Adapt* |
| 03 | Heat* |
| 04 | Season* |
| 05 | 01 or 02 |
| 06 | 03 or 04 |
| 07 | 05 and 06 |
| 08 | 05 and 06. Limiters – Medicine |
| 09 | 05 and 06. Limiters – Medicine. Health professions. |
| 10 | 05 and 06. Limiters – Medicine. Health professions. Narrow by language - English |
| 11 | 05 and 06. Limiters – Medicine. Health professions. Narrow by language – English. Document type – article |
| Final. | ( ( TITLE-ABS-KEY ( acclim* ) ) OR ( TITLE-ABS-KEY ( adapt* ) ) ) AND ( ( TITLE-ABS-KEY ( heat* ) ) OR ( TITLE-ABS-KEY ( season* ) ) ) AND ( LIMIT-TO ( LANGUAGE , "English" ) ) AND ( LIMIT-TO ( SUBJAREA , "MEDI" ) OR LIMIT-TO ( SUBJAREA , "HEAL" ) ) AND ( LIMIT-TO ( DOCTYPE , "ar" ) ) |

**Table S3.** Search Strategy.

| **Database:** Cochrane Library | |
| --- | --- |
| **Order** |  |
| 01 | Acclim* |
| 02 | Adapt* |
| 03 | Heat* |
| 04 | Season* |
| 05 | 01 or 02 |
| 06 | 03 or 04 |
| 07 | 05 and 06 |

Table S4. Modified McMaster Critical Review Form for Quantitative Studies

CITATION:

|  |
| --- |
|  |
|  |

Comments

| **Study concept** | **Question** | **Scoring (awarded points)**  **No = high risk of bias**  **Yes = low risk of bias** |
| --- | --- | --- |
| **Study Purpose:**  Was the purpose stated clearly? | Outline the purpose of the study. How does the study apply to the research question? Was relevant background literature reviewed? | - No (0) - Yes (1) - Unsure (0) |
| **Study Design (1):**  Was there any control within the study design? | Describe the study design. Was the design appropriate for the study question? | - No (0) - Yes (1) - Unsure (0) |
| **Study Design (2):**  Was there any randomization? |  | - No (0) - Yes (1) - Unsure (0) |
| **Sample:**  Was the sample described and the size justified? | Sampling (who; characteristics; how many; how was sampling done?) If more than one group, was there similarity between the groups? Was this sample size justified? | - No (0) - Yes (1) - Unsure (0) |
| **Reliability and Validity:**  Were the outcome measures reliable and valid? | Was the frequency of the outcome measurements identified? | - No (0) - Yes (1) - Unsure (0) |
| **Intervention:**  Intervention was described in detail? | Provide a short description of the intervention (focus, who delivered it, how often, setting). Could the intervention be replicated? | - No (0) - Yes (1) - Unsure (0) |
| **Statistical Analysis:**  Were the  analysis method(s) appropriate? And reported in terms of statistical significance? | What were the results? Were they statistically significant (i.e., p < 0.05)? If not statistically significant, was study big enough to show an important difference if it should occur? If there were multiple outcomes, was that considered for the statistical analysis? | - No (0) - Yes (1) - Unsure (0) |
| **Conclusion:**  Conclusions were appropriate given study methods and results | What did the study conclude? What are the implications of these results? What were the main limitations or biases in the study? | - No (0) - Yes (1) - Unsure (0) |
|  | Total score: | /8 |

Modified version of an extracted file from: Evidence-Based Practice Research Group at McMaster University. Quantitative Review Form. Copyright: Law M, Stewart D, Pollock N, Letts L, Bosch J and Westmorland M, 1998. McMaster University. Available from: http://srs-mcmaster.ca/research/evidence-based-practiceresearch-group/#OlXEXdby.
